# Annotations

**Published:** 1904-04-30

**Authors:** 


### ANNOTATIONS.

### The Midwives Act.

At the annual meeting last year of the Birmingham and District General Practitioners' Union, a resolution was adopted to the effect that the Council should formulate rules for the guidance of members in respect to attendance on midwives' cases. We now learn, through the courtesy of the *British Medical Journal*, that the following report of the Ethical Committee of the Council has been read and approved :—"The question of medical attendance in midwives' cases having been thoroughly discussed, the committee considers that it is impossible to formulate a code of rules for the guidance of members. They wish to point out :—(1) That the relation of medical men to the public is not altered by the Midwives Act, either financially or professionally. (2) That a larger fee should be charged in midwives' cases. (3) That when summoned to a case by a midwife, a medical practitioner's relation to the patient is the same as if summoned by the patient."

This report is opposed to a resolution which was passed last year by one branch of the Birmingham and District General Practitioners' Union to the effect that the members of that branch should agree not to attend midwives' cases at a fee of less than one guinea, and that the midwife concerned in the case would be held responsible for obtaining the fee. As we had occasion to point out in a leading article in our issue of the 12th ult., the medical practitioner has certain moral responsibilities to fulfil, and one of these responsibilities is to succour the lying-in woman who is in peril. In carrying out this obligation the medical man is doing a public service, and from the public authorities he ought to seek his reward if it be not forthcoming from the individual who has benefited at his hands. Any indirect methods of extorting payment by threats directed at the midwife in attendance might have deplorable results by encouraging hesitation on her part to call in timely medical assistance through fear of injury should any difficulty over the doctor's fee subsequently arise.

### Management of Scotch Poorhouses.

The Departmental Committee of the Local Government Board of Scotland which has been engaged in an inquiry into the methods and conditions under which Poor-law medical relief is administered in Scotland has now issued its report with a series of suggested administrative reforms. It is allowed that as compared with the year 1845, when an organised system of medical relief was first introduced, great improvements have been effected in the care and treatment of the sick poor, both indoor and outdoor. But it is also noted that experience shows the necessity for reform in certain details of administration. Among other proposals the committee recommends that the powers of medical officers should be enlarged, and advises that in some cases the salaries paid to medical officers call for revision ; that the appointment of a medical officer in each parish should be required by statute and that in certain instances a dwelling-house should be provided for him ; and that the position of out-door medical officers should be placed upon a statutory footing. Concerning the supply of medicines the committee is opposed both to the inclusion of this as an obligation covered by the salary of the medical officer, and also to the practice of providing medicines by a fixed annual payment made either to the medical officer or a druggist ; it is suggested that in outlying districts parochial depôts for medicines should be established. Pauper nurses, it is advised, should be abolished and a general system of trained nursing introduced ; probationers might be attracted to the poorhouses by the payment of a salary. In reference to the patients the committee recommends a more varied dietary and that the routine use of stimulants should be checked ; that where the inmates are few certain combinations might be effected ; and that much of the unoccupied space in the poorhouses might, with slight alteration, be profitably used in the treatment of pulmonary phthisis. This last recommendation is one of very wide interest. The Poor-law infirmaries undoubtedly offer an opportunity for determining on a large scale the true value of the open-air treatment of tuberculosis. We think that most of the recommendations of the committee will command the sympathy of the medical profession, and certainly not the least valuable is that which recognises the Poor-law infirmaries as offering an occasion for applying modern therapeutic methods in the treatment of pulmonary tubercle.

### The Sociological Society.

The institution of a society intended to form a common ground for all those whose studies and work are concerned with "the national and civil history of man, his achievements and his ideals" is an event which should interest alike the man of science, the philosopher, and the social reformer. The various fields of observation which are included in the programme of the newly-established Sociological Society have been without question industriously cultivated by many individual workers, but there is wanting an opportunity for comparison and co-ordination, and this opportunity it is hoped will now be afforded. It is somewhat remarkable that the country which can claim Herbert Spencer as its distinguished son has among its universities not a single professor of sociology, whilst the importance and significance of this subject have received wide academic recognition in France, Germany, and the United States. The mere admission that good and capable men have again and again directed their efforts for social reform along lines proved by experience to be misleading, is sufficient in itself to show that there is at present a very imperfect apprehension of the essential conditions of the problem for which a solution is so much desired. Here, as in many other departments of human activity, zeal without knowledge has proved helpless and even disastrous. The scientific method appears slow and callous to those who insist upon prompt and immediate salvation; but it has the sanction of success in many directions. An effort to apply it on a large and organised scale to social problems ought to call for the support of all good citizens and more especially of those who, like the practitioner of medicine, are aware of the essential failure of many modern proposals. It is satisfactory to know that not only has the Sociological Society come into existence but also that it has already secured considerable encouragement.

